# The Office of Coroner

**Published:** 1886-11

**Authors:** 


### THE OFFICE OF CORONER.

The able and exhaustive article on the subject of "Lex Coronaria," written for THE JOURNAL by Hooper A lexander, Esq., and published in our August number, 1885, was so generally well received and favorably noticed by our contemporaries in this country and England as to inspire the belief that it is the common opinion of the profession that the office of coroner fitly and properly belongs to the medical profession. The people of Fulton county, a few years since, gave expression to this opinion by the election of Dr. Drake, of this city, whose able and faithful discharge of the duties of the office illustrates the value of professional training for the position.

No one who has not received a medical education, let him be ever so intelligent in other respects, is competent, from the very nature of the case, to perform its duties.

Were the public justly to appreciate the functions of the coroner, and did they reflect how greatly the due administration of justice in criminal cases depends upon the preliminary investigations of that officer, one would suppose that none other than an intelligent, medically educated man would ever be tolerated. The public have, however, hitherto judged differently, and the examination of the most intricate questions, involving character and life, has been committed to the jurisdiction of men profoundly ignorant of everything relating to those questions. That this is a great public evil cannot be questioned, and the correction of it ought to be attempted as speedily as possible.

No legislative act can of course touch the case, and it only remains for the united profession to exercise their influence in awakening the public mind to a just appreciation of the subject.

